# Integrating molecular profiling into glioma diagnosis: implications of the WHO-CNS5-2021 classification of adult-type diffuse gliomas in Colombian patients

**DOI:** 10.3389/fneur.2025.1691983

**Published:** 2026-01-06

**Authors:** Omar Echeverría, Andrés Felipe Patiño-Aldana, Ana María Chinchilla, Nora Contreras, Stevan Peralta, Nicolas Caballero, Hanna Valentina Tovar, José Manuel Palacio, Verónica Uribe, Matteo Mineo-Pachón, Fernando Velandia, William Mauricio Riveros Castillo, Nattaly Valero, Daniel Felipe Silgado-Guzmán, Alejandro Ondo-Mendez, Dora Janeth Fonseca-Mendoza

**Affiliations:** 1 Center for Research in Genetics and Genomics (CIGGUR), School of Medicine and Health Sciences, Institute of Translational Medicine (IMT), Universidad del Rosario, Bogotá, Colombia; 2 Clinical Research Group, School of Medicine and Health Sciences, Universidad del Rosario, Bogotá, Colombia; 3 Consultant, Bogotá, Colombia; 4 Department of Neurosurgery, Hospital Universitario Mayor Méderi and Samaritana University Hospital, School of Medicine and Health Sciences, Universidad del Rosario, Bogotá, Colombia; 5 Department of Molecular Diagnosis, Genética Molecular de Colombia SAS, Bogotá, Colombia

**Keywords:** 1p/19q co-deletion, brain neoplasms, gliomas, IDH, mutations, TERT promoter, WHO CNS5

## Abstract

**Introduction:**

Gliomas are the most frequent type of primary malignant central nervous system (CNS) tumors, representing a group of heterogeneous neoplasms with variable clinical behavior that require adequate diagnostic accuracy. The identification of molecular biomarkers has recently gained significance for the diagnosis, prognosis, and treatment of CNS tumors; the application of current clinical guidelines is necessary. Our study performed a molecular characterization of gliomas in a cohort of Colombian patients using the recommendations of the 2021 World Health Organization (WHO) CNS 5 classification.

**Materials and methods:**

We analyzed 22 Colombian patients with CNS tumors. Molecular techniques including Sanger sequencing, multiplex ligation-dependent probe amplification (MLPA) and methylation-specific MLPA (MS-MLPA) were used to identify mutations in *IDH1*, *IDH2*, *TERT*, and *EGFR*, as well as 1p/19q co-deletion and *MGMT* promoter methylation status.

**Results:**

Our results demonstrated a 23% discordance rate between histopathologic and molecular classifications, with most of the discrepancies due to an initial histopathologic classification of glioblastomas, which were molecularly reclassified as astrocytomas. In addition, molecular profiling allowed us to identify non-canonical mutations, including *IDH1* p.R132S, which has shown an impact on patient prognosis.

**Discussion:**

We highlight the importance of incorporating molecular methods to improve diagnostic accuracy and achieve personalized treatments for gliomas, as proposed by the current 2021 WHO CNS 5 tumor classification guidelines. Performing new studies with larger patient cohorts integrating clinical data is necessary to determine the behavior, epidemiology, and therapeutic outcomes of this type of tumor more comprehensively.

## Introduction

Gliomas are the most common primary malignant brain tumors in adults, representing a heterogeneous group of neoplasms with significant variability in clinical behavior, response to treatment, and overall prognosis. Adult-type diffuse gliomas account majority of gliomas and comprise three types of tumors: oligodendrogliomas, which are *IDH*-mutant and 1p/19q co-deleted, having the best prognosis; *IDH*-mutant astrocytomas with an intermediate outcome; and *IDH*-wildtype glioblastomas, which are associated with poor prognosis (1, 2). Historically, the classification and diagnosis of gliomas has been based on their histopathological characteristics. However, advances in molecular genetics have revolutionized our understanding of these tumors, leading to the identification of key molecular markers that influence both the biological behavior of gliomas and patient outcomes (3).

In this context, the molecular genetics of gliomas is one of the most challenging and dynamic areas of modern oncology. Over the past decade, extensive genomic profiling studies have uncovered distinct molecular subtypes of gliomas, which have profound implications for diagnosis, prognosis, and treatment stratification (4). Single-gene targets, including mutations in genes such as *EGFR*, *IDH*, and *TP53*, have been extensively studied because of their roles in glioma pathogenesis (5).

Key genomic alterations, such as mutations in the *IDH1* and *IDH2* genes, co-deletion of 1p/19q, and *MGMT* promoter methylation, among others, have shifted the paradigm from a purely histological classification to an integrated approach that combines both molecular and histopathological data. The World Health Organization’s (WHO) updated classification of central nervous system tumors in 2016, with further revisions in 2021, reflects this shift and formally recognizes the critical role of molecular characterization in glioma diagnosis (6).

The molecular heterogeneity of gliomas not only aids in refining diagnostic categories but also provides valuable prognostic information. For instance, *IDH*-mutant gliomas are associated with more favorable outcomes than their *IDH*-wildtype counterparts, which typically exhibit more aggressive clinical behavior (7). Moreover, the identification of these molecular alterations has opened the door to targeted therapeutic strategies aimed at improving survival and quality of life for glioma patients (5).

Advances in precision medicine therapies for cancer, particularly glioblastoma, are promising. Expanding research in this area could improve the prognosis and current situation for glioblastoma patients (8).

In this study, we explored the impact of molecular characterization of gliomas in Colombian patients by utilizing molecular techniques, such as multiplex ligation-dependent probe amplification (MLPA), Sanger sequencing, and methylation analysis through methylation-specific MLPA (MS-MLPA). Our study allowed us to establish the frequency of point mutations and gene copy number variations (CNVs), including *IDH1*, *IDH2*, *TERT* and *EGFR*, as well as the analysis of 1p/19q co-deletion. These findings enabled the application of the WHO 2021 molecular classification algorithm and to evaluate the discordance rate between histopathological and molecular diagnosis.

## Materials and methods

### Sampling and data collection

This study included 22 patients who underwent surgical resection of brain tumor tissue following the physician’s recommendation. The samples were obtained from three neurosurgical referral centers located in Bogotá, Colombia. Eligible patients were invited to participate, and those who agreed signed informed consent. The study included patients over 18 years of age who underwent neurological surgery with suspected diffuse glioma. Patients with other types of cerebral malignancy such as atypical meningiomas were excluded. Patient demographic data, including age, sex and body mass index (BMI) were extracted from clinical records when available. The clinical classification of the glioma type was determined by a consultant neuropathologist. All the analyses were approved by the Ethics Committee of Universidad del Rosario. This study adhered to the guidelines of the Declaration of Helsinki and Ethical Guidelines from the Council For international Organizations of Medical Sciences (Approval DVO005 1299-CV1142).

### Molecular analysis

#### Multiplex ligation-dependent probe amplification

Genomic DNA was obtained from brain tumor tissue of 22 patients using the QuickDNA^™^ Miniprep Plus Kit (Zymo Research). MLPA was performed using the commercial kit SALSA MLPA P105 Glioma-2, version D3. The P105 Glioma-2 kit contained 56 MLPA probes, with amplified products ranging from 126 to 500 nucleotides. It includes 43 probes for CNV analysis of *PDGFRA*, *EGFR*, *CDKN2A*, *PTEN*, *CDK4*, *MIR26A2*, *MDM2*, *NFKBIA*, and *TP53* genes, and 13 reference probes.^1^

Similarly, the SALSA MLPA P088 Oligodendroglioma 1p-19q kit, version D1 (MRC-Holland, Amsterdam), was used to detect the 1p-19q co-deletion. This kit contained 59 MLPA probes with amplified products ranging from 126 to 509 nucleotides, including 19 probes for 1p and 12 probes for 19q. Following the WHO glioma classification criteria (2021) and described by Bertero et al. (9), co-deletion analysis was performed in patients with *IDH* mutations.

Following the manufacturer’s recommendations for each MLPA reaction, 50 ng of DNA was denatured in a thermocycler for 5 min at 98 °C. After cooling to 25 °C, the probemix and MLPA buffer were added to each sample, mixed, and incubated for 1 min at 95 °C, followed by hybridization for 16.5 h at 60 °C. The ligation reaction was performed by incubating the ligase-65 mix at 54 °C, followed by heating to 98 °C for 5 min. Finally, PCR using universal-tagged primers was conducted with 35 amplification cycles (95 °C for 30 s, 60 °C for 30 s, and 72 °C for 1 min), followed by incubation at 72 °C for 20 min. Amplification products were separated by capillary electrophoresis on an automatic 3500 genetic analyzer, using GeneScan 500 Liz as a molecular line marker. DNA extracted from tumor tissues of two patients with meningiomas was used as negative control. Because meningiomas differ from gliomas in both cellular origin and molecular biology, these samples were included as controls. Additionally, blood samples from individuals without a family history of brain tumors were used as normal controls for all MLPA processes.

#### Methylation-specific MLPA

MS-MLPA was employed to analyze the methylation profile of the O6-methylguanine DNA methyltransferase (*MGMT*) gene. The SALSA MLPA Probemix ME012 MGMT-IDH-TERT, version B1, was used, containing 31 MS-MLPA probes with amplified products between 123 and 317 nucleotides. Eight MS-MLPA probes included at least one recognition site for the HhaI restriction enzyme, enabling methylation status assessment. This probemix also featured six specific mutation probes that identified the most frequent point mutations in *IDH1* (p.R32H and p.R132C), *IDH2* (p.R172K and p.R172M), and the *TERT* promoter region (C228T and C250T). Additionally, 13 reference probes, unaffected by HhaI enzyme digestion, served as normal controls for methylation status analysis.

#### Cell culture for MS-MLPA

The human glioblastoma cell line T98G was used as a positive methylation control. T98G cells were cultured in Dulbecco’s Modified Eagle’s Medium (DMEM) supplemented with 10% fetal bovine serum and 100 U/mL penicillin/streptomycin at 37 °C in a 5% CO_2_ atmosphere. DNA extraction from T98G cell culture was performed using the QuickDNA^™^ Miniprep Plus Kit (Zymo Research). MS-MLPA was performed using 50 ng of DNA from each patient and cell culture, following the manufacturer’s recommendations.^2^

#### Sanger sequencing for identification of mutations in *IDH1*, *IDH2*, and *TERT* promoter region

Flanking regions of exon 4 of *IDH1* and *IDH2* were amplified by polymerase chain reaction (PCR). Amplification primer sequences were as follows: *IDH1*: F: 5′-tagttctctttgtagttggcaccc-3′/R: 5′-ctacacc cattaagcaaggtatgaa-3′ and *IDH2*: F: 5′-tcatgaagaattttaggacccccg-3′/R: 5′ ccatcctcttgtctctgcagtac-3′. PCR conditions were as follows: initial denaturation at 95 °C for 10 min; 30 cycles of 95 °C for 40 s, 60 °C for 40 s, and 72 °C for 40 s; and final extension at 72 °C for 10 min. For mutations C228T and C250T in the *TERT* gene located in the promoter region, a hemi-nested PCR was performed. Primer sequences and PCR conditions were as described by da Costa et al. (10) PCR products were visualized on 1.2% agarose gels stained with ethidium bromide. The amplified products were analyzed using Sanger sequencing methodology.

### Data analysis

#### MLPA analysis

MLPA analysis for CNV identification in *PDGFRA*, *EGFR*, *CDKN2A*, *PTEN*, *CDK4*, *MIR26A2*, *MDM2*, *NFKBIA* and *TP53* genes, and the 1p-19q co-deletion was performed using Coffalyser-Net software.^3^ Data generated with SALSA MLPA P105 and P088 were normalized intra-sample and inter-sample. The number of copies was determined using the dosage quotient distribution (DQ) value. Patient status was determined based on the following relationships: DQ = 0 (homozygous deletion); 0.40 < DQ < 0.65 (heterozygous deletion); 0.80 < DQ < 1.20 (normal); 1.30 < DQ < 1.65 (heterozygous duplication); 1.75 < DQ < 2.15 (homozygous duplication), all other values (ambiguous result). DQ values followed MRC-Holland recommendations.

Methylation analysis using MS-MLPA was performed using Coffalyser-Net software. The methylation percentage in patient samples, normal controls, and T98G cell line were determined by comparing each signal in the digestion reaction with the corresponding signal in the undigested reaction.

#### Sanger sequencing analysis

Sanger sequencing was used to determine the presence or absence of mutations in the *TERT* promoter and exon 4 of *IDH1* and *IDH2* genes. Reference sequences from Ensembl were analyzed using FinchTV v.1.5.0 (Geospiza Inc.). Alignment with wildtype sequences was conducted using Clustal W v2.1.^4^ The effects of molecular variants on protein were determined using Expasy Translate.^5^ Descriptive frequency analyses were performed for all identified molecular variants in the population.

### Molecular classification

Finally, a molecular classification of patient samples was performed according to the guidelines established by the World Health Organization (WHO, 2021), applying the algorithm described by Bertero et al. (9). We evaluated the proportion of sample reclassified among histopathological and molecular diagnosis.

### Molecular description

The frequencies of the methylation states and mutations identified by MS-MLPA, MLPA, and Sanger sequencing were determined. It is important to note that the *TERT* promoter mutations and 1p/19q co-deletion were not analyzed in 100% of the cases. For *TERT*, the analysis was only conducted in patients with *IDH* wildtype, while the 1p/19q co-deletion was exclusively assessed in IDH-mutant cases, following the recommendations of WHO 2021 glioma classification (9).

## Results

### Sociodemographic characteristics

A total of 22 patients were included in the study. Of these, 40.9% (*n* = 9) were male, 54.54% (*n* = 12) were female, and 1 case was unknown. Additionally, histopathological diagnoses were distributed as follows: glioblastoma 72.7% (*n* = 16), astrocytoma 13.6% (*n* = 3), oligodendroglioma 9% (*n* = 2), ganglioglioma 4.5% (*n* = 1) (Table 1).

**Table 1:** Demographic characterization of patients (*n* = 22).

Characteristic	*n* (%)
Gender	Male: 9 (40.9%)
	Female: 12 (54.54%)
	Unknown: 1 (4.54%)
Age	<50 years: 8 (36.36%)
	>50 years: 11 (50%)
Histopathology classification	
Glioblastoma	16 (72.7%)
Astrocytoma	3 (13.6%)
Oligodendroglioma	2 (9%)
Ganglioganglioma	1 (4.5%)

### Molecular analysis

#### Sanger sequencing for identification of mutations in the *IDH1*, *IDH2*, and *TERT* promoter regions

Of the total samples analyzed (*n* = 22), 40.90% (*n* = 9) presented pathogenic variants in *IDH1*: p.R132H in 27.27% (*n* = 6) and p.R132S in 13.63% (*n* = 3). Additionally, 13.63% (*n* = 3) presented p.G105G synonymous variant, whereas for *IDH2*, only 4.54% (*n* = 1) presented the p.R172W variant (in a sample also harboring p.R132H). Following the WHO CNS-5 classification criteria, the *TERT* promoter was evaluated in the *IDH* wildtype samples via Sanger sequencing, of which 38.46% (5 out of 13) presented the C228T variant, while the remaining samples did not present either C228T or C250T.

#### Multiplex ligation-dependent probe amplification

**Figure 1:**
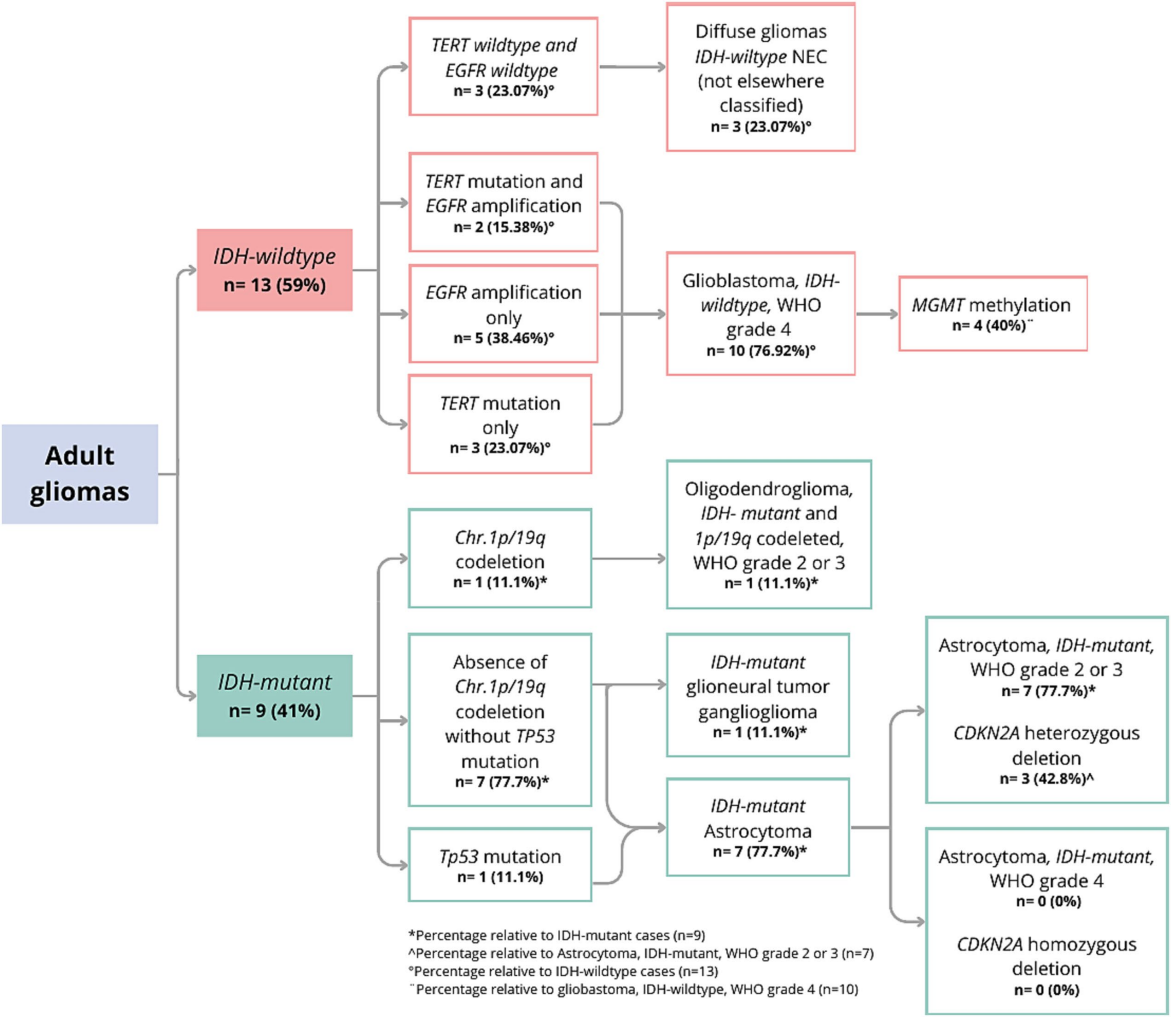
Molecular classification with WHO CNS-5 adapted gliomas flowchart.

The analysis of CNVs showed that 13.63% (*n* = 3) of the samples had *PDGFRA* amplification, 36.36% (*n* = 8) had *EGFR* amplification, 22.72% (*n* = 5) had homozygous *CDKN2A* deletion, and 31.81% (*n* = 7) had heterozygous deletions. All homozygous deletions of *CDKN2A* were observed in patients with *IDH*-wildtype tumors. Regarding *PTEN*, 27.27% (*n* = 6) of samples had a heterozygous deletion, whereas 4.54% (*n* = 1) had a homozygous deletion. Finally, 9.09% (*n* = 2) had *TP53* duplication, and 13.63% (*n* = 3) had *CDK4* amplification. During the analysis of the 1p/19q co-deletion, it was observed that 11.11% (1 out of 9) of the *IDH*-mutant samples displayed this alteration (Figure 1).

#### Methylation-specific multiplex ligation-dependent probe amplification

*MGMT* analysis revealed that 54.54% (*n* = 12) of the samples exhibited hypermethylation in the promoter region of this gene. Since the SALSA MS-MLPA Probemix contains probes that can identify *IDH1* (p.R132H = c.395G>A and p.R132C = c.394C>T), *IDH2* (p.R172K = c.515G>A and p.R172M = c.515G>T), and *TERT* (C228T and C250T), we compared these findings with the Sanger sequencing methodology, finding a 100% concordance. However, samples identified by MLPA with allelic fractions below 50% showed very low sequencing peaks. In this context, we estimated that MLPA is more sensitive for identifying IDH1 mutations. This behavior was also observed for *TERT* mutations.

### Molecular classification

The WHO CNS-5 classification was performed based on the *IDH* genotype (9). Figure 1 shows the results of the molecular classification of the analyzed patients. A total of 40.9% (9 out of 22) of the samples were classified as IDH-mutant. In *IDH1*, the variants p.R132H and p.R132S were identified, with frequencies of 27.27 and 13.63%, respectively. One of the patients (GLIO 23) presented a double mutation in the *IDH1* and *IDH2* genes (p.R132H and p.R172W). In 59% of the cases (13 out of 22), no variants were identified in *IDH* (IDH-wildtype). Among the *IDH*-mutant samples, 11.1% (1 out of 9) had the 1p/19q co-deletion and were classified as “Oligodendroglioma, *IDH*-mutant and 1p/19q co-deleted, WHO grade 2 or 3.” One *IDH*-mutant sample had a histopathological diagnosis of ganglioglioma; therefore, it was not reclassified using the diffuse glioma algorithm. Instead, we limited our analysis of this sample to describing its molecular profile. For the remaining 7 samples, the absence of the 1p/19q co-deletion supported the classification as “Astrocytoma, IDH-mutant.” Upon evaluating the *CDKN2A* gene of these samples, it was found that 42.8% (3 out of 7) had a heterozygous deletion, while none had a homozygous deletion, and thus they were classified as “Astrocytoma, IDH-mutant, WHO grade 2 or 3.” Interestingly, only one of the three *IDH*-mutant samples with heterozygous *CDKN2A* deletion also harbored a *TP53* duplication.

In the analyzed population, 59% of the patients were *IDH*-wildtype (13 out of 22). Of these, 76.9% (10 out of 13 *IDH*-wildtype) presented mutations in *TERT* and/or *EGFR* amplifications. Specifically, 50% (5 out of 10) presented *EGFR* amplification, 30% (3 out of 10) had the *TERT* C228T mutation, and 20% (2 out of 10) presented both molecular variants. None of the evaluated samples harbored the *TERT* C250T mutation. These molecular findings allowed the classification of the tumors as “Glioblastoma, IDH-wildtype, WHO grade 4,” of which 40% (4 out of 10) had *MGMT* promoter hypermethylation. Interestingly, 3 out of 13 gliomas did not present mutations in *IDH*, *TERT*, or *EGFR*, and were thus classified as “Diffuse gliomas IDH-wild type NEC (Not Elsewhere Classified)” (Figure 1).

### Comparison between histopathological and molecular classification

**Figure 2:**
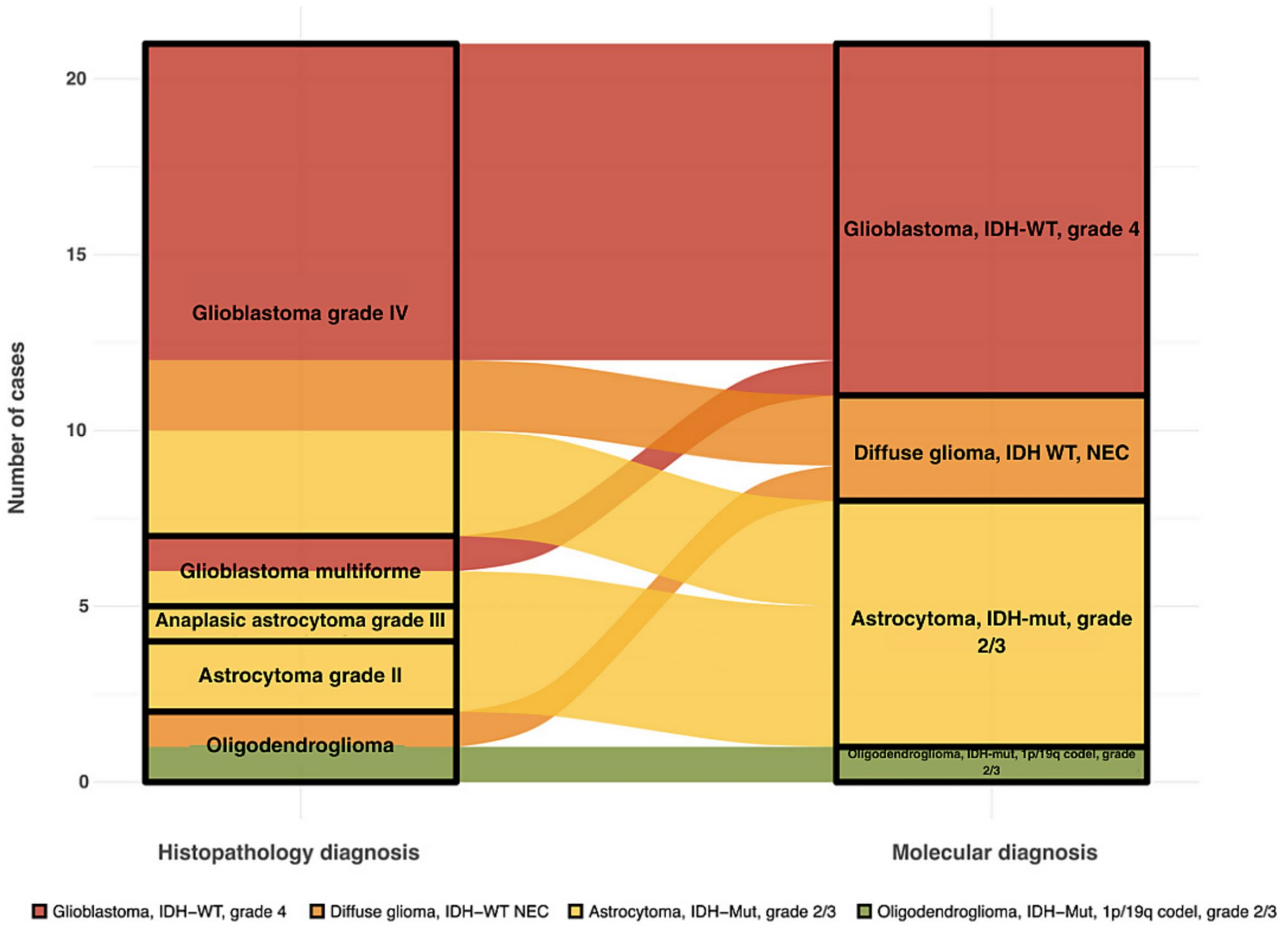
Alluvial plot showing shifts between histopathological and molecular diagnosis in adult diffuse gliomas.

The comparison between histopathological and molecular classifications revealed a discordance rate of 23%, indicating discrepancies between traditional pathology-based diagnoses and molecular-based reclassification. Diagnostic shifts were mainly due to the reclassification of glioblastoms as astrocytomas based on *IDH* mutation status (Figure 2). Specifically, four samples (GLIO 1, GLIO 7, GLIO 23, GLIO 227) initially classified as glioblastomas based on histopathology were reclassified as “Astrocytoma, *IDH*-mutant, WHO grade 2 or 3” according to molecular analysis (Table 2). This difference highlights the limitations of histopathological classification alone, particularly in cases where molecular features provide a more accurate prognosis and treatment guidance.

**Table 2:** Comparison between histopathology classification and molecular classification.

Sample	Histopathology classification	Molecular classification
GLIO 001	Glioblastoma, WHO grade 4	Astrocytoma, *IDH-mutant*, WHO grade 2 or 3
GLIO 002	Glioblastoma multiforme	Glioblastoma, *IDH-wildtype*, WHO grade 4
GLIO 003	Glioblastoma, WHO grade 4	Glioblastoma, *IDH-wildtype*, WHO grade 4
GLIO 004	Oligodendroglioma	Oligodendroglioma, *IDH-mutant* and 1p/19q codeleted, WHO grade 2 or 3
GLIO 007	Glioblastoma, WHO grade 4	Astrocytoma, *IDH-mutant*, WHO grade 2 or 3
GLIO 008	Glioblastoma, WHO grade 4	Glioblastoma, *IDH-wildtype*, WHO grade 4
GLIO 009	Glioblastoma, WHO grade 4	Glioblastoma, *IDH-wildtype*, WHO grade 4
GLIO 012	Glioblastoma, WHO grade 4	Glioblastoma, *IDH-wildtype*, WHO grade 4
GLIO 017	Glioblastoma, WHO grade 4	Glioblastoma, *IDH-wildtype*, WHO grade 4
GLIO 020	Astrocytoma	Astrocytoma, *IDH-mutant*, WHO grade 2 or 3
GLIO 021	Anaplastic ganglioglioma	NA^*^
GLIO 022	Glioblastoma, WHO grade 4	Glioblastoma, *IDH-wildtype*, WHO grade 4
GLIO 023	Glioblastoma, WHO grade 4	Astrocytoma, *IDH-mutant*, WHO grade 2 or 3
GLIO 024	Glioblastoma, WHO grade 4	Glioblastoma, *IDH-wildtype*, WHO grade 4
GLIO 200	Glioblastoma, WHO grade 4	Diffuse glioma, IDH-wildtype NEC
GLIO 227	Glioblastoma multiforme	Astrocytoma, *IDH-mutant*, WHO grade 2 or 3
GLIO 639	Anaplastic astrocytoma	Astrocytoma, *IDH-mutant*, WHO grade 2 or 3
GLIO 638	Astrocytoma grade II	Astrocytoma, *IDH-mutant*, WHO grade 2 or 3
GLIO 665	Glioblastoma, WHO grade 4	Glioblastoma, *IDH-wildtype*, WHO grade 4
GLIO 738	Glioblastoma, WHO grade 4	Glioblastoma, *IDH-wildtype*, WHO grade 4
GLIO 757	Oligodendroglioma	Diffuse glioma, IDH-wildtype NEC
GLIO 915	Glioblastoma, WHO grade 4	Diffuse glioma, IDH-wildtype NEC

NA^*^: reclassification not applied.

Notably, no discordance was observed in the classification of astrocytomas or oligodendrogliomas when both the histopathological and molecular criteria were applied. Furthermore, certain diagnoses in pathology reports, such as “Anaplastic Astrocytoma,” are not part of the current WHO classification for adult diffuse gliomas, underscoring the evolving nature of glioma classification as molecular profiling becomes more integrated into clinical practice.

## Discussion

Integrating molecular profiling to characterize gliomas is essential for achieving an accurate diagnosis. Using advanced molecular techniques, we demonstrated a 23% disagreement rate between histopathological diagnosis alone and molecular diagnosis according to the WHO CNS 5 classification. The discrepancies were due to histopathological classifications of glioblastoma, whereas molecular classification identified *IDH*-mutant astrocytoma, WHO grade 2–3. The implications of an integrated diagnosis extend well beyond purely taxonomic considerations. Appropriate diagnosis determines the prognosis, feasible chemotherapeutic strategies and personalized emerging targets. For instance, the median survival rate for *IDH*-mutant astrocytomas ranges from 9 to 11 years for grades 2 and 3, respectively (11). In contrast, for glioblastoma, the most common primary CNS malignant neoplasm, is less than 2 years (2).

Consistent with global literature, we found a high frequency of IDH wildtype glioblastoma in our cohort, representing 59% (13 out of 22) of cases (12). The origin of this tumor type is believed to result from the accumulation of somatic mutations in neural stem cells and glial precursor cells, which confer selective growth advantages and uncontrolled proliferation (13). In addition to its high frequency, glioblastomas are a one of the main fields of research in neuro-oncology due to their poor prognosis, rapid progression, and challenging therapeutic management (14).

IDH-mutant astrocytomas were the second most common subtype in our sample according to molecular classification representing 31% (7 out of 22) of the cases. This tumor type was observed in seven of the analyzed patients, and interestingly, three of them were younger than 50 years as reported in previous studies (2). Astrocytomas generally have a better prognosis and treatment response compared to glioblastoma. However, these are slow-growing tumors which can progress to more aggressive forms over time (15).

Only one patient (4.5%) had an oligodendroglioma IDH-mutated 1p/19q co-deletion. These are well-differentiated tumors that arise from oligodendrocytes and are associated with favorable prognosis. Similar to our findings, other authors have indicated that oligodendrogliomas are a relatively rare primary brain tumor, accounting for approximately 5% of all brain tumors (16–19). This patient was 50 years old, which is consistent with the reported incidence peak of 30 to 50 years, with an estimated survival of up to 20 years (20).

Distinguishing IDH-mutant from IDH wildtype gliomas is crucial for the accurate classification of adult diffuse gliomas. IDH mutations result in the production of the oncometabolite D-2-hydroxyglutarate (D-2HG), which is associated with reduced levels of NADPH and glutathione, thereby promoting apoptosis through oxidative stress. This condition is associated with more favorable prognoses and improved responses to temozolomide (TMZ) therapy (21, 22). Our study identified that the most frequent mutation in *IDH1* was p.R132H (27.3%), and previous studies in Colombian patients reported frequencies ranging from 34 to 57%. These differences might be explained by the heterogeneity of glioma subtypes included and the sequencing methods used (23, 24). Interestingly, in other populations worldwide, p.R132H accounts for over 90% of detected mutations in IDH-mutant diffuse gliomas, suggesting potential population-specific differences in mutation frequency, underscoring the importance of conducting studies in understudied populations, such as Latin American individuals (25, 26).

In this regard, our study demonstrated a higher frequency of non-canonical *IDH1* (p.R132S) mutations as a proportion of the IDH-mutant gliomas, compared to an European population (34% vs. 10%) (26). Non-canonical mutations have been associated with better survival outcomes (HR 0.47, 95% CI, 0.28–0.81) compared to *IDH* canonical mutations, despite lower 1p/19q co-deletion rates. Non-canonical mutations could modify the enzymatic activity of IDH, and variations in the 2-DHG concentration might lead to differences in DNA methylation levels (27). Interestingly, in one patient carrying this mutation (*IDH1* p.R132S), a copy number variation (CNV) affecting *TP53*, consisting of a heterozygous duplication, was identified. Although heterozygous duplications of *TP53* are rare events, they may co-occur with *IDH1* mutations, a combination that is generally associated with worse clinical outcomes, although the magnitude and biological context of this effect depend on the tumor type and the allelic status of *TP53* (28, 29). Importantly, the 2-DHG produced by mutant *IDH* alters α-ketoglutarate-dependent chromatin regulators and can disrupt p53-dependent transcriptional programs. In addition, *IDH1*-mutant cells exhibit p53-mediated senescence phenotypes that influence therapeutic response (30). This molecular context underscores the importance of considering the biological and therapeutic impact of double-mutant tumors (*IDH1* plus *TP53* alterations) in clinical decision-making. Additionally, although this patient exhibited a heterozygous *CDKN2A* deletion, its clinical impact is expected to be limited, as hemizygous *CDKN2A* deletions have been reported not to significantly worsen overall survival or progression-free survival in IDH-mutant astrocytomas and oligodendrogliomas, CNS WHO grades 2 and 3 (31).

Additionally, 13.6% of the patients exhibited the SNP rs11554137 p.G105G, located in the region of the gene where the most frequent somatic *IDH* mutations occur. This synonymous variant, located in exon 4, was reported by Acquaviva et al. (32) to have a frequency three times higher in patients diagnosed with infiltrating gliomas compared to the normal population. However, this finding has not been replicated in other association studies have not replicated this finding, and to date, its impact on prognosis remains inconclusive (33).

Our findings are clinically relevant and demonstrate the impact of molecular analysis compared to pathology studies based solely on immunohistochemistry and the exclusive detection of *IDH1* p.R132H, which limits the identification of protein expression for other mutations (23).

Regarding *IDH2* mutations, our study aligns with other reports indicating lower frequencies of molecular variants of this gene. Although both enzymes are involved in energy metabolism, IDH1 is primarily located in the cytosol and peroxisomes, whereas IDH2 is in the mitochondria. Although *IDH1* and *IDH2* mutations are typically mutually exclusive (34), and despite the infrequency of variants in both genes, which did not exceed 0.4% (35), we identified a case with co-existing *IDH1* R132H and *IDH2* R172W mutations. Yuile et al. (36) recently described a patient with concurrent *IDH1* R132H and *IDH2* R172G mutations who experienced a favorable response to treatment, suggesting that coexisting mutations may positively influence the prognosis. However, the implications of such co-occurrence have not been clearly defined. Proper genotyping of both *IDH1* and *IDH2* is necessary to achieve the correct diagnosis, prognosis and therapeutic options involving IDH mutant inhibitors (37). Further studies in underrepresented populations are required to understand the frequency and biological and clinical implications of this co-occurrence (38).

Our extended molecular analysis allowed us to identify deletions in *CDKN2A*, which are crucial for determining the prognosis and treatment options for patients with astrocytomas. When CDKN2A loses its functionality owing to deletions, cell cycle control mediated by p16 and p14ARF proteins is disrupted. In 37.5% (3 out of 8) of the IDH-mutated astrocytoma patients, a heterozygous deletion was found, compromising its tumor suppressor function. This finding contrasts with previous reports in other populations, in which approximately 22% of IDH-mutant astrocytomas were estimated to harbor homozygous deletions of *CDKN2A* (39).

Although homozygous deletion of *CDKN2A* was adopted by the WHO 2021 classification as a defining molecular feature of grade 4 IDH-mutant astrocytomas, this alteration has also been extensively described in IDH-wildtype gliomas, where it is associated with a more aggressive tumor biology and shorter survival, and may therefore serve as an independent poor prognostic biomarker (40). In our analysis, all homozygous *CDKN2A* deletions were identified exclusively in patients with IDH-wildtype tumors. A meta-analysis including 714 patients demonstrated that the median overall survival of patients with IDH-wildtype gliomas harboring homozygous *CDKN2A* deletion was 13.0 months, compared with 18.0 months in patients without the deletion (*p* = 0.014, log-rank test) (41, 42). In addition, single-institution series and treatment-adjusted analyses have corroborated this adverse effect, showing that *CDKN2A* deletion is an independent biomarker associated with worse overall survival (OS) and progression-free survival (PFS), with a multivariable hazard ratio of approximately 1.57 in cohorts of IDH-wildtype glioblastoma (40).

In IDH-wildtype adult diffuse gliomas, *TERT* promoter and *EGFR* amplifications are glioblastoma-defining events, even without necrosis or microvascular proliferation. These mutations play a key role in the prognosis of this highly aggressive tumor. *TERT* promoter mutations increase telomerase activity, allowing for indefinite tumor cell replication, while *EGFR* amplifications lead to continuous activation of intracellular signaling pathways that promote cell proliferation. Hotspot analysis of *TERT* promoter showed that 50% of our patients presented the *TERT* p.C228T mutation, located in the promoter region at position −124, but none had de C250T variant. Given that the C228T mutation is present in ~75% of TERTp mutated gliomas, it is not surprising that we did not identify the C250T mutation in our sample (43). These mutations create new binding sites for transcription factors of the E-26 family, leading to increased mRNA transcription of the telomerase catalytic subunit, which confers radiotherapy resistance (44).

*MGMT* gene promoter methylation is especially valuable for patient assessment due to its high predictive value for treatment response (45, 46). We observed *MGMT* gene promoter hypermethylation in 40% of glioblastomas using MS-MLPA. *MGMT* encodes a DNA damage repair enzyme that is silenced by hypermethylation of its promoter, conferring chemosensitivity to alkylating agents, such as TMZ. It has also been proposed as an early biomarker useful to differentiate real progression from pseudo-progression with a good sensitivity of 80% but a poor specificity (67%), which leads to uncertainty given the high proportion (~30%) of false positives, raising concerns about its clinical utility for defining the requirements for further therapy (45).

Patient-reported outcomes, such as health-related quality of life, might be affected not only by cancer symptoms but also by treatment side effects. Suitable treatment selection is a major concern for optimizing health outcomes in patients with glioma and depends on the correct diagnosis. For instance, clinical follow up after surgery might be a viable option for young patients with IDH-mutant astrocytoma grade 2 without adverse prognostic factors. When further therapy is needed, combined radio and chemotherapy are recommended over either alone. Procarbazine, lomustine and vincristine are recommended options for low grade adult diffuse gliomas over alkylating drugs such as TMZ (47, 48). However, TMZ chemoradiotherapy, which is currently used for high-grade gliomas, may have a lower toxicity profile and a similar efficacy. A head-to-head comparison of radiotherapy plus TMZ or rocarbazine, lomustine and vincristine is currently ongoing (NCT00887146).

Moreover, the role of *IDH* mutations in tumorigenesis has led to the emergence of targeted therapies. Vorasidenib, an IDH-1 and IDH-2 inhibitor improved the progression free survival (27.7 months vs. 11.1 months) compared to placebo in patients with residual or recurrent grade 2 oligodendroglioma or astrocytoma in a phase III trial. Further therapies targeting downstream phenomena of IDH mutations, such as histone and DNA hypermethylation, DNA synthesis or repair mechanisms, and immune activation by an *IDH1*-R132H specific vaccine are under evaluation. Currently, only *BRAF* pV600E evaluation for recurrent or progressive CNS tumors has been proved useful as a clinical standard for therapy selection (49).

The molecular analysis of gliomas and their classification using the updated 2021 WHO guidelines provide a valuable tool for accurate diagnosis, prognosis, and therapeutic decision-making. However, the sensitivity and specificity of the molecular techniques used to identify mutations present technical challenges. Current methodologies such as MLPA, Sanger sequencing, digital PCR, and nanopore sequencing represent alternatives for molecular diagnostics (50–52).

In this study, we used MLPA and Sanger sequencing to identify mutations in *IDH1*, *IDH2*, and *TERT*, thereby revealing the advantages and disadvantages of these methods. In the case of *IDH1* and *IDH2*, MLPA was limited to detecting only four specific point variants (R132H, R132C, R172M, and R172K), which would have missed the identification of the *IDH1* p.R132S and *IDH2* p.R172W variants, representing 18.2% of the IDH variants in our population. From this perspective, Sanger sequencing allows for the identification of all mutations present in this region; however, it has been estimated that this methodology has limitations in detecting somatic variants with allele frequencies below 15% (53).

Regarding *TERT* promoter mutations, we obtained more reliable results using MLPA, which detected positive signals between 32 and 280%, indicating a good sensitivity to low-frequency somatic mutations. Sanger sequencing presents technical difficulties, because the most frequent mutations occur in GC-rich regions and require a mutant allele frequency of at least 20% to generate a positive signal (10).

This technique surpasses other methods, such as methylation-specific PCR, which has high false-positive and false-negative rates due to methylation heterogeneity, reducing its utility for clinical decision-making (54). Similarly, immunohistochemistry assays show reduced reliability due to high inter-observer variability and lack of correlation with clinical outcomes (46).

Many challenges arise in targeted therapies, such as tumor heterogeneity, neuronal-glia growing-promoting interactions, escape mechanisms and immunosuppressive microenvironment, among others. However, a limiting step, especially in scarce resource settings, might be the adoption of the molecular characterization and its integration in clinical practice to properly diagnose and care for each patient, while generating updated clinical and epidemiological information of the different glioma types and grades. There is a lack of information about the adherence to WHO CNS 5 classification in limited resource settings, and some authors have questioned the practical benefits and applicability due to cost issues of this classification. Molecular classification possibly increases the gap in care of brain tumor patients between high and low and middle income countries (55).

Our study has some limitations. First, the limited sample size might contribute to an uncertainty about the reclassification rate and practical implications of molecular classification. Second, we reported uniquely molecular based classifications, rather than the recommended layered diagnosis which could lead to misclassification bias, especially of Not Elsewhere Classified tumors. Third, chromosome 7 gain and chromosome 10 loss (+7/−10) and histone isoform status were not evaluated; therefore, a definitive classification of patients with *IDH*, *TERT*, and *EGFR*-wildtype tumors cannot be established. However, our results established the genetic profile of gliomas in a sample of Colombian patients, following the WHO 2021 recommended algorithm. Although several studies in Colombia have performed mutation identification, gene expression analysis, and clinical characterization, to the best of our knowledge, this is the first study to conduct molecular classification incorporating all genetic hallmarks required by the WHO 2021 for adult diffuse gliomas. Our findings underscore the need to incorporate molecular methodologies for accurate tumor classification, which would allow precise diagnosis, and prognosis. While targeted treatment remains elusive but available for selected cases, is a main field of research within the framework of precision medicine.

In this context, our exploratory study demonstrates the clinical utility of applying molecular studies in accordance with WHO recommendations. However, we estimate that a larger cohort of patients and comprehensive clinical data would be necessary to validate our findings on genetic profiles and molecular reclassification.

## Conclusion

This study highlights the importance of molecular classification in adult diffuse gliomas, following the WHO 2021 guidelines, and demonstrates the power of genomic profiling to improve diagnostic accuracy. The discrepancies between molecular and histopathological classifications underscore the need to integrate molecular tools into routine diagnostics, as this approach significantly affects clinical decision making. These findings support the growing role of molecular techniques in precision medicine, providing a pathway for more personalized treatments and improved outcomes for glioma patients. Future studies with larger cohorts and integrated clinical data are necessary to fully explore the potential of molecular diagnostics in neuro-oncology.

## Data Availability

The original contributions presented in the study are included in the article/supplementary material, further inquiries can be directed to the corresponding author.

## Footnotes

1
https://mrcholland.com/product/P105/1484

2
https://mrcholland.com/product/ME012/367

3
https://mlpa.com

4
http://clustal.org/

5
https://expasy.org/

## Glossary

CNS: Central nervous system
WHO: World Health Organization
MLPA: Multiplex ligation-dependent probe amplification
MS-MLPA: Methylation-specific multiplex ligation-dependent probe amplification
CNVs: Gene copy number variations
BMI: Body mass index
DMEM: Dulbecco’s Modified Eagle’s Medium
TMZ: Temozolomide
D-2HG: D-2-hydroxyglutarate
PVC: Procarbazine, lomustine and vincristine
PCR: Polymerase chain reaction
*IDH*: Isocitrate dehydrogenase
*TERT*: Telomerase reverse transcriptase
*EGFR*: Epidermal growth factor receptor
*MGMT*: Methylguanine-DNA methyltransferase
*TP53*: Tumoral protein 53
*PDGFRA*: Platelet-derived growth factor receptor, alpha
*CDKN2A*: Cyclin-dependent kinase inhibitor 2a
*PTEN*: Phosphatase and tensin homologue
*CDK4*: Cyclin-dependent kinase 4
*MIR26A2*: Micro RNA 26a2
*NFKBIA*: Nuclear factor kappa-b inhibitor, alpha
